# Supplementary material for: Circulating Tumor Cells as a Tool of Minimal Residual Disease Can Predict Lung Cancer Recurrence: A longitudinal, Prospective Trial
Source: Diagnostics (Basel). 2020 Mar 6;10(3):144. doi: 10.3390/diagnostics10030144 (PMC7151004; doi:10.3390/diagnostics10030144)
Supplement: Supplementary file 1 [file diagnostics-10-00144-s001.pdf]

Supplementary Figures

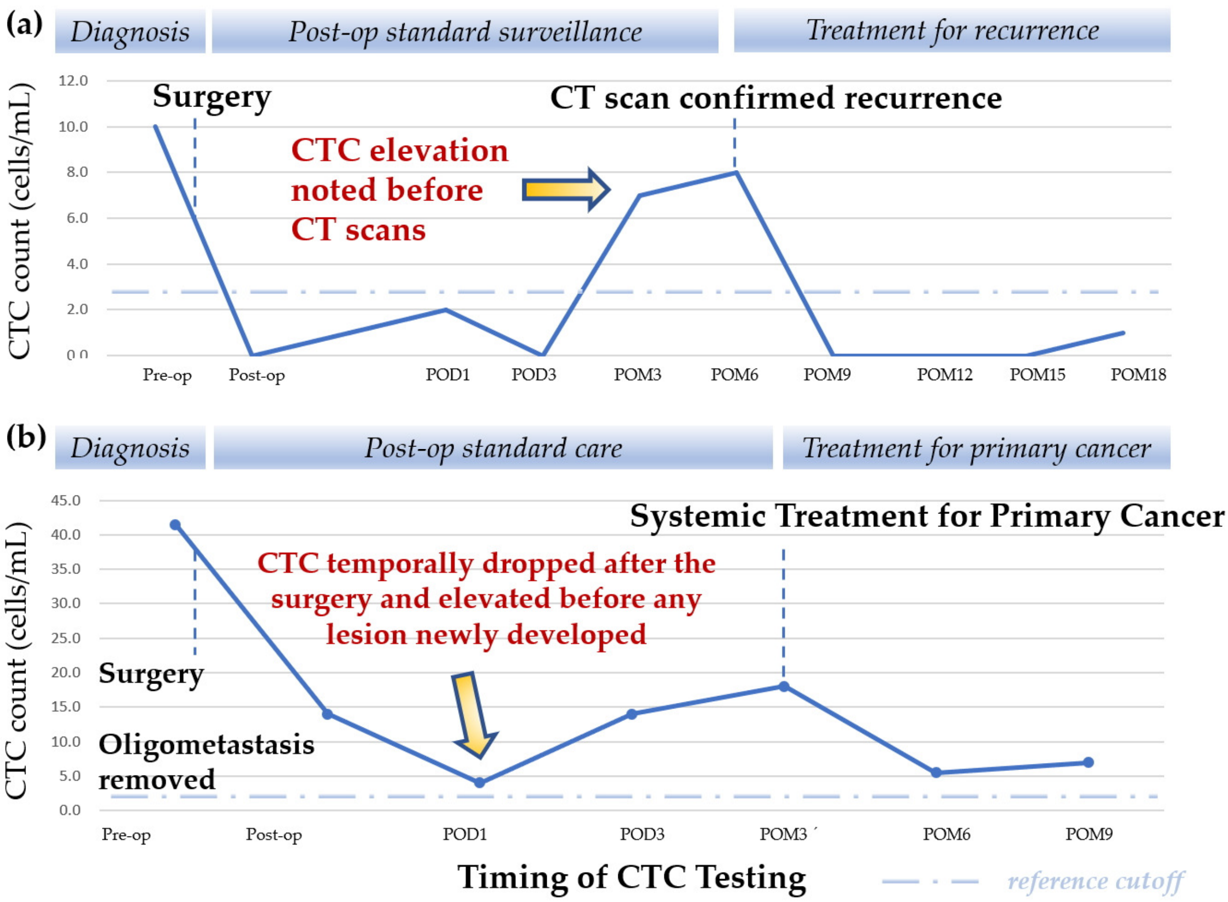

Figure S1. Demonstrations of Possible Role of CTC testing in lung cancer and pulmonary oligometastasis. (a) shows a representative case of CTC testing predicting the recurrence from the primary lung cancer. (b) displays the CTC dynamics during surveillance in a patient later proven to be an oligometastasis from another solid tumor.

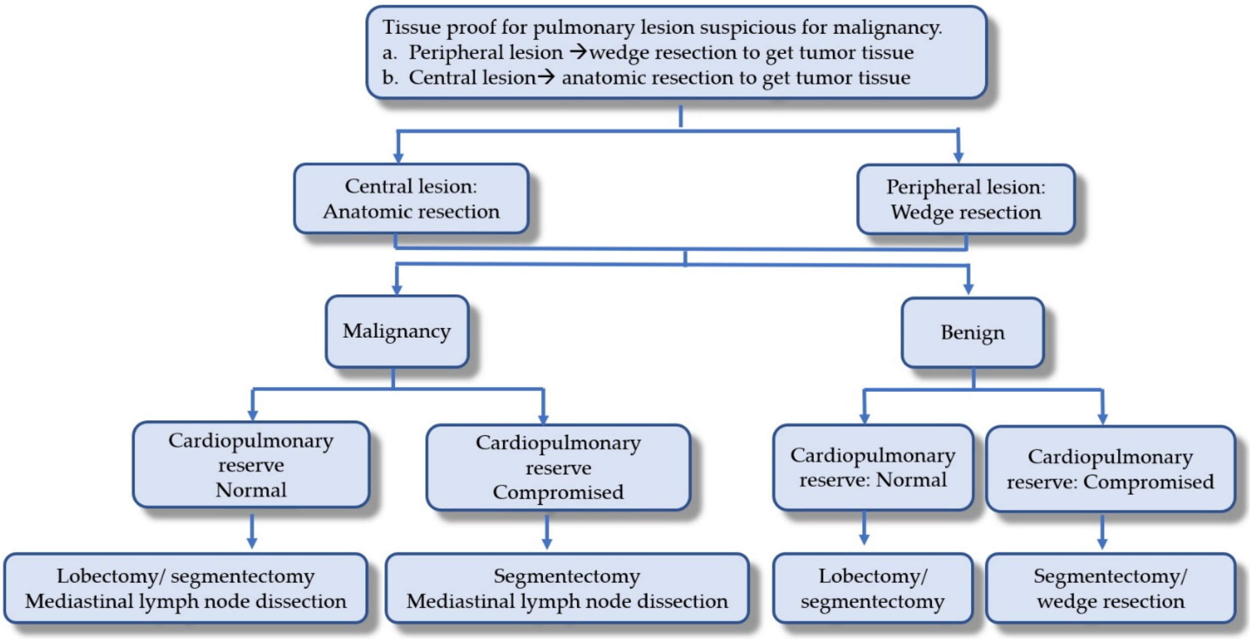

Figure S2. The algorithm of surgical management for pulmonary lesions with a suspicion of malignancy.

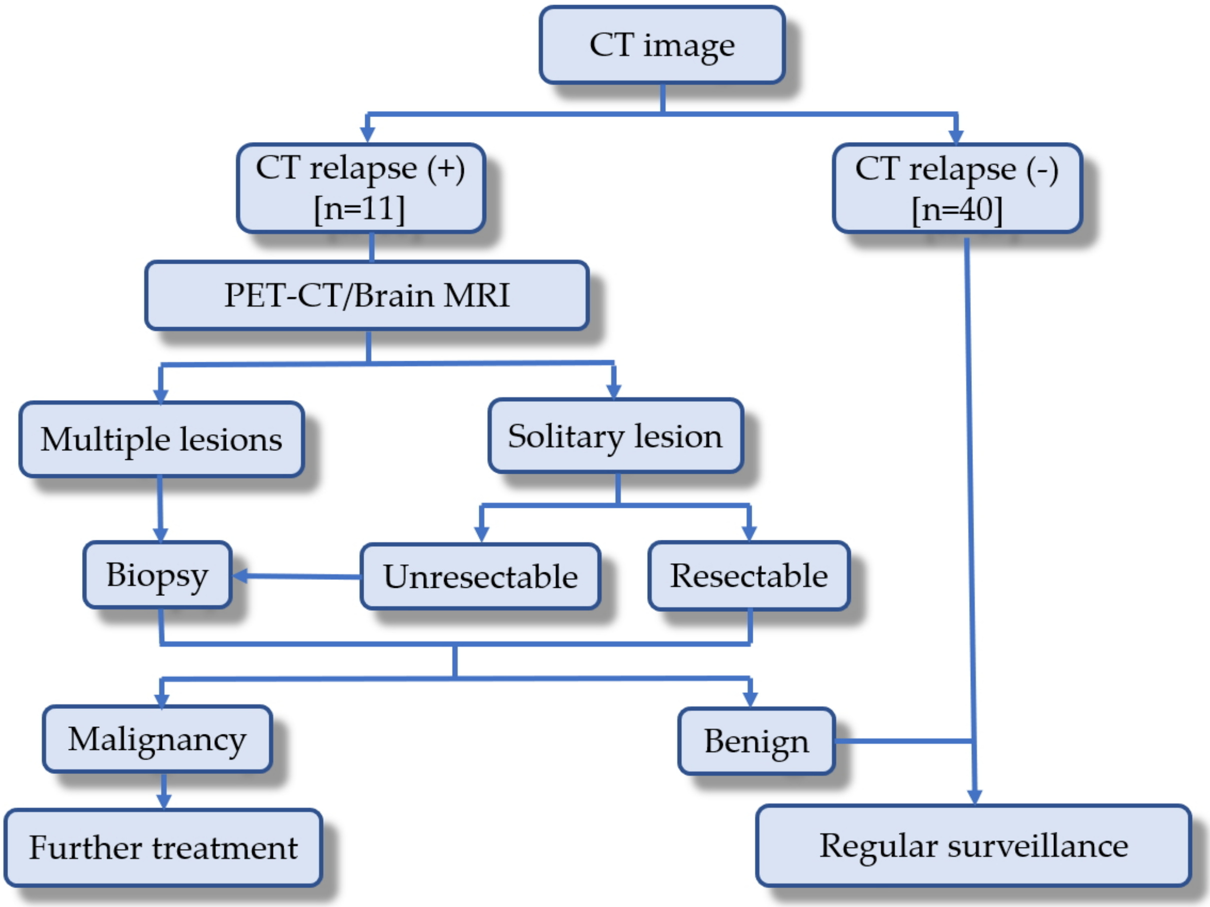

Figure S3. Surveillance algorithm and corresponding management for possible relapse lesions. (Abbreviations: CT, computed tomography; PET, positron emission tomography; MRI, Magnetic Resonance Imaging.).
